# Supplementary material for: MetaRibo-Seq measures translation in microbiomes
Source: Nat Commun. 2020 Jun 29;11:3268. doi: 10.1038/s41467-020-17081-z (PMC7324362; doi:10.1038/s41467-020-17081-z)
Supplement: Supplementary file 10 — Supplementary Data 7 [file 41467_2020_17081_MOESM10_ESM.zip › File2/Confidence_High_Taxonomy/357623_out.krona.html]

Javascript must be enabled to view this page.

members
magnitude
magnitudeUnassigned
count
unassigned
taxon
rank

357623\_out

24

superkingdom
2
24

phylum
976
24

200643
24
class

171549
24
order

171552
24
family

838
23
genus

165179
21
species

SRS012989\_contig\_number\_6564SRS013098\_contig\_number\_18106SRS017375\_contig\_number\_2SRS018888\_contig\_number\_2722SRS019285\_contig\_number\_488SRS024132\_contig\_number\_22901SRS044535\_contig\_number\_11916SRS047044\_contig\_number\_5287SRS047433\_contig\_number\_7848SRS049712\_contig\_number\_1734SRS053398\_contig\_number\_2271SRS077641\_contig\_number\_13SRS104036\_contig\_number\_3665SRS1041134\_contig\_number\_859SRS1041137\_contig\_number\_25504SRS143181\_contig\_number\_237SRS143876\_contig\_number\_24453SRS144297\_contig\_number\_4102SRS144506\_contig\_number\_18944SRS144603\_contig\_number\_36SRS144714\_contig\_number\_5694

2
59823

SRS097958\_contig\_number\_17933SRS150029\_contig\_number\_2469
species

genus
1
577309

species

SRS015782\_contig\_number\_15335
454154
1
